# Surgery after Failed Transcatheter Aortic Valve Implantation: Indications and Outcomes of a Concerning Condition

**DOI:** 10.3390/jcm11010063

**Published:** 2021-12-23

**Authors:** Mohamed Salem, Christina Grothusen, Mostafa Salem, Derk Frank, Mohammed Saad, Markus Ernst, Thomas Puehler, Georg Lutter, Assad Haneya, Jochen Cremer, Jan Schoettler

**Affiliations:** 1 Department of Cardiovascular Surgery, Campus Kiel, University Hospital Schleswig-Holstein, 24105 Kiel, Germany; christina.grothusen@uksh.de (C.G.); markus.ernst@uksh.de (M.E.); Thomas.Puehler@uksh.de (T.P.); Georg.Lutter@uksh.de (G.L.); assad.haneya@uksh.de (A.H.); Jochen.Cremer@uksh.de (J.C.); Jan.Schoettler@uksh.de (J.S.); 2 Department of Cardiology and Angiology, Campus Kiel, University Hospital Schleswig-Holstein, 24105 Kiel, Germany; mostafa.salem@uksh.de (M.S.); derk.frank@uksh.de (D.F.); Mohammed.Saad@uksh.de (M.S.); 3 DZHK (German Centre for Cardiovascular Research), Partner Site Hamburg/Kiel/Lübeck, Potsdamer Str. 58, 10785 Berlin, Germany

**Keywords:** TAVI degeneration, SAVR after TAVI, long-term outcome of TAVI

## Abstract

Objectives: The number of transcatheter aortic valve implantations (TAVI) has increased enormously in recent decades. Transcatheter valve prosthesis failure and the requirement of conventional surgical replacement are expected to attract more focus in the near future. Indeed, given the scarcity of research in this field, the next decade will likely represent the beginning of a period of meaningful exploration of the degenerative changes that occur with transcatheter valves. The current study represents—through a series of consecutive cases—one of the first analyses of the underlying causes of TAVI failure, i.e., degenerative, functional and infective, followed by surgical aortic valve replacement (SAVR) and postoperative outcome. Methods: Between October 2008 and March 2021, 2098 TAVI procedures, including 1423 with transfemoral, 309 with transapical, and 366 with transaortic access, were performed in our institution. Among these, 0.5% (number(*n*) = 11) required acute SAVR (*n* = 6) within 7 days (*n* = 3) or later (*n* = 2), and were included in the study. Results: Valve stent dislocation was the most common cause of replacement (83%). Causes of replacement within 7 days after TAVI were multifactorial. In the later course, endocarditis was the sole indication for SAVR after TAVI. TAVI with transapical or transaortal approach had a higher EuroSCORE II (10.9 (7.2–35.3) vs. 3.5 (1.8–7.8)). Their 30-day mortality after surgical conversion was higher (67% vs. 20%), when compared to those who underwent a transfemoral procedure. The longest documented survival beyond 30 days was 58 months. Conclusions: The causes of SAVR after TAVI failure are multifactorial, and include biological, physical and infectious factors. An acceptable midterm prognosis may be expected in patients with physical causes when dislocation of the catheter prosthesis is observed; in such cases, emergency conversion is required. Conversion due to infection, as in cases of endocarditis, had the worst outcome. Prognosis after conversion due to degeneration is still problematic, due to a lack of autopsies and the recent history of prosthetic implantations.

## 1. Introduction

Due to demographic changes, the incidence of aortic valve stenosis requiring treatment is increasing. While approximately 15,000 isolated aortic valve procedures were performed in Germany in 2010, nearly 25,000 were performed in 2019 [1]. For decades, the gold standard for the treatment of symptomatic aortic valve stenosis was conventional surgical aortic valve replacement (SAVR) alone [2]. Since 2006, transcatheter aortic valve implantation (TAVI) has been considered a well-established alternative technique [3]. In 2017, for the first time, more than half of isolated aortic valve procedures in Germany were performed as TAVI, and in 2019, the TAVI proportion was already around 60% [1].

According to current recommendations, TAVI is the procedure of choice for older and sicker patients, i.e., those aged 75 years and older with a Society of Thoracic Surgeons Score (STS Score) or EuroSCORE II of at least 4% [4]. TAVI application in younger patients with fewer comorbidities is still being investigated in clinical trials [5,6]. If possible, TAVI is performed via the transfemoral approach (TF-TAVI) [4]. In cases of small diameters of femoral arteries, peripheral arterial occlusive disease and severe atherosclerosis or marked kinking of the descending aorta, TAVI can also be applied via the left ventricular apex (TA-TAVI) or the ascending aorta (TAO-TAVI), as well as transsubclavian and transcarotid, each using a minimally invasive technique [7,8].

The results after TAVI are promising. The increasing expertise of heart teams and ongoing improvements of catheter valve implants have made TAVI safer as a low-complication procedure. Even the number of paravalvular leaks, which were seen more frequently in the past, is decreasing [9]. The durability of the implants appears to be sufficient, despite initial concerns [10]. However, the degenerative, biomedical and infectious factors leading to TAVI dysfunction have not yet been thoroughly investigated. Until now, TAVI durability data are based on the absence of reintervention or post-TAVI-SAVR in populations of elderly patients with an already a low survival rate of 30% at 5 y [11,12]. This is due to the recent history of prosthetic implantation. Moreover, the criteria of choosing TAVI patients such as old age and high comorbidities, and thus, the associated higher mortality rate, do not facilitate accurate analyses of the causes of the degenerative changes and failure of transcatheter prostheses.

In this paper, we report our experience regarding the various factors leading to TAVI dysfunction and the need for subsequent surgical replacement.

## 2. Materials and Methods

### 2.1. Patients

Between October 2008 and March 2021, 2098 consecutive TAVI procedures, including 1423 with transfemoral, 309 with transapical, and 366 with transaortic access, were performed in the cardiovascular department of the University Hospital of Schleswig-Holstein, Campus Kiel. TAVI was performed either through balloon-expandable or self-expandable valves. Retrospectively, we reviewed all patients who underwent surgical replacement of aortic valve following TAVI. The study population was defined as any SAVR after TAVI due to any form of deterioration of the primary implanted transcatheter aortic valve (TAV). The causes included malposition or dislocation of the TAV, paravalvular regurgitation, degeneration of TAV, annulus and ventricular perforation or infective endocarditis. SAVR were done either as an emergency procedure after unsuccessful primary TAVI or due to a prosthesis failure after successful TAVI. The cases were diagnosed either through transthoracic echocardiography or transesophageal echocardiography intra- or postoperative or through postoperative computer tomography. Data were collected and extracted from the institution’s database and from medical records. The study protocol was approved by the local ethics committee in Kiel (D 415/21), and patient consent was obtained prior to and during hospital stay.

### 2.2. Statistical Analysis and Definitions

Descriptive statistics are reported as mean ± SD for normally distributed continuous variables or otherwise as median and 25th–75th percentile (interquartile range). Absolute and relative frequencies are reported for categorical variables. A univariate comparison between the groups for categorical variables was made using the x² and the Fisher’s exact test. A survival analysis was performed with the Kaplan–Meier method through extraction from the city database. The normality of continuous variables was assessed by Kolmogorov-Smirnow-Test. A statistical analysis was performed using the SPSS Statistics software (Version 18.0) and Stata 10 SE (Stata Corp., College Station, TX, USA). The primary endpoint was 30-day mortality. Secondary endpoints were intraoperative variables and postoperative course (e.g., ventilation time, bleeding, acute renal failure, neurological complications and late mortality).

### 2.3. Surgical Technology

All operations were done either as emergency procedures (such in cases of dislocation of the TAV, annulus rupture or coronary ostium occlusion during the primary TAVI procedure), or either on an urgent or elective basis (such in cases of late deterioration of valve function due to high grade paravalvular leak or endocarditis). Surgery was performed via median sternotomy using cardiopulmonary bypass and cardioplegic cardiac arrest. Salvage of the dislocated valve stents into the distal ascending aorta or the aortic arch sometimes required circulatory arrest and deep hypothermia. A transverse supracoronary aortic incision was made to remove dislocated valve stents and to visualize the aortic root. In SAVR cases after initially successful TAVI, the deployed wire meshes of the transcatheter valve were separated from the aortic wall with a dissector, and the prosthetic stent was then removed piece by piece, using a wire cutter if necessary. Finally, after excision of the aortic valve leaflets and careful decalcification of the aortic valve annulus, implantation of a biological prosthetic heart valve was performed in the usual manner. In cases of annulus rupture, a biological patch was used to strengthen the aortic wall.

## 3. Results

### 3.1. Demographic Data and Preoperative Variables

Between October 2008 and March 2021, 2098 TAVI procedures, including 1423 with transfemoral, 309 with transapical, and 366 with transaortic access, were performed in our institution. Among these, 0.52% (*n* = 11; five patients (45%) transfemoral TAVI (TF-TAVI), six patients (55%) transaortal or apical TAVI (TA/TAO-TAVI)) of patients underwent SAVR after TAVI failure, of which six were done on an emergency basis, three within 7 days, and two at a later time. The clinical characteristics of this population are shown in Table 1. The median age was 79 years (interquartile range (64–85) and four (36%) were female. The mean EuroSCORE II was 7.8 (1.8–35.3). Ten patients (91%) suffered from coronary heart disease with previous coronary stenting. Four patients (36%) required dialysis and seven (64%) presented with peripheral arterial disease (PAD). When comparing the preoperative demographic data between TF-TAVI und TA/TAO TAVI, we found that patients with surgical TAVI were more often male (83% vs. 40%), had a higher EuroSCORE II (median 10.9 (7.2–35.3) vs. 3.5 (1.8–7.8)), and tended to have more chronic obstructive lung disease (83% vs. 20%), renal insufficiency with dialysis (50% vs. 20%), and peripheral artery disease (83% vs. 40%), Table 1.

### 3.2. Pro-Procedual TAVI-Metrics

TAVI valve sizing and design were based on annulus dimension (mainly diameter), as well as the distance between aortic valve annulus and both sinotubular junction and the right and left ostia. The important measurements are represented in Table 2. Calcium score was mainly assessed as ordinary CAC-Score, and in recent cases, was assessed through absolute Agatston—Score, Table 2.

### 3.3. Type of Prosthesis and Sizes of Implanted TAV

The ratio between self-expanding to balloon-expanding prostheses was 55% vs. 45%. The sizes ranged from 23 to 34 mm with no noticeable difference between the groups. Table 3 provides information on the prosthesis types and sizes used in previous TAVI procedures.

### 3.4. Core Data and Indication of SAVR

An analysis of the core data of the 11 patients showed that all subjects received a biological prosthetic heart valve, i.e., 64% received a porcine aortic valve and 36% a bovine pericardial tissue heart valve. One patient was stabilized with an extracorporeal circulatory support system (ECLS) even before SAVR. In another patient, closure of a newly formed ventricular septal defect (VSD) was required in addition to SAVR. The indication for surgical intervention SAVR was dislocation of TAV in five patients, either in LVOT or in the ascending aorta or aortic arch. One patient suffered from moderate to severe paravalvular leakage. Endocarditis as the cause of intravalvular regurgitation was documented in two patients. Two patients suffered from annulus perforation (one with VSD). Occlusion of the coronary left main trunk was a reason for SAVR in one patient. Late TAV degeneration was recognized at scheduled echocardiographic or computer tomography follow-up; see Table 4.

### 3.5. Timing of Surgery

In six cases (55%), a single-stage conversion was required. Three patients (27%) underwent SAVR at 7 days after the TAVI procedure, and two (18%) at an interval of more than 3 months after TAVI. Single-stage surgery (80% vs. 33%) and dislocation of the catheter valve (80% vs. 17%) were observed more frequently in the group which had undergone transfemoral procedure, Table 5.

### 3.6. Intraoperative Variables

Procedual durations in cases of transfemoral or surgical TAVI did not differ significantly. A preference for a specific biological valve prosthesis could not be determined. Implant sizes did not differ in our comparison; see Table 6.

### 3.7. Postoperative Variables

Patients who underwent SAVR after a failed surgical TAVI had a longer duration of mechanical ventilation (median 121 (48–283) vs. 28 (15–125)) and appeared to require more frequent postoperative dialysis (67% vs. 20%), associated with a longer stay in the intensive care unit (median 9.5 (2–26) vs. 3 (2–6)). Their 30-day mortality was higher than that of the group which had undergone the primary transfemoral procedure (67% vs. 20%), Table 7.

Survival at the time of follow-up ranged from 1 to 58 months. Two patients are currently still alive. In total, a cumulative survival of nearly 10 patient-years has been achieved to date.

## 4. Discussion

At present, the use of TAVI is increasing in comparison to SAVR. TAVI is currently more applicable in medium- and in lower-risk patients, rather than only high-risk patients. This brings about a need for more adequate studies and strategies to be implemented, as not taking action regarding these young patients—as opposed to older, multimorbid patients—is no longer an option. The circumstances of TAVI failure, including etiology, incidence, management and outcome, are still under analysis. The causes of these failures should be more thoroughly analyzed in the near future. Thus, our study aimed to analyze the various early and late factors leading to TAVI failure.

Moreover, it is not clear whether patients after TAVI should undergo SAVR in emergency unsuccessful TAVI or in the course after primarily successful TAVI. In this context, there are only registry data for emergency cardiac surgical procedures during TF-TAVI [13,14]. Both TAVI surgeries with transapical or transaortic access and two-stage surgeries later in the course were not considered in this registry. Furthermore, these registries did not focus in detail on the factors leading to TAVI failure.

The present study considers almost 13 years of TAVI experience. From 28 October 2008 to 31 January 2021, 2098 TAVI procedures were performed in Kiel, including 1423 TF-TAVI and 675 surgical TAVI, of which 309 were transapical and 366 were transaortic. During this time, a total of only 11 patients (0.5%), i.e., six with primarily unsuccessful TAVI and five after primarily successful TAVI, underwent SAVR early-postoperatively for prosthesis-associated complications or because of subsequent prosthesis failure.

The causes of TAVI failure are multifactorial. According to the literature, during TF-TAVI, left ventricular perforation by the guidewire, annulus rupture, embolization or migration of the transcatheter valves, and aortic dissection are prominent as emergency indications for conversion to cardiac surgery [13]. These problems are generally associated with the TAVI, and consequently, are also observed in surgical implantations. In our work, a performed SAVR was considered an inclusion criterion, regardless of whether there the transfemoral or surgical approach was applied. Cases in which cardiac perforations were treated using a heart–lung machine, or even other cardiac surgical procedures without explantation of the TAVI prosthesis, such as isolated replacement of the ascending aorta for Stanford type A aortic dissection, were not included in our work. Therefore, the reason for primary failure of the TAVI procedure in our collective was mainly dislocations of the catheter valve. Only one of our patients required emergency conversion to SAVR for a different reason, namely, occlusion of the main left coronary trunk by the implanted valve stent.

Indication for cardiac surgery in the early-postoperative phase was due to to a periannular rupture, an annulus-near ventricular septal defect, and a catheter valve that was not fully deployed with increasing paravalvular leakage. All procedures in the early course after primary successful TAVI were required within 7 days of the procedure. The complications observed during this period were among the known intraprocedural complications. It is plausible that there are TAVI-associated problems that present with a latency of days in individual cases. According to the European Registry on Emergency Cardiac Surgery during TAVI, more than 90% of complications still occur during the TAVI procedure. Only about 3% of TAVI patients manifest problems requiring emergency cardiac surgery with a median sternotomy after more than 24 h [13].

Later, only two patients underwent SAVR because of manifest endocarditis of the valve stent. Endocarditis is not a TAVI-specific problem. In general, patients undergoing valve replacement are at higher than average risk of endocarditis. Prognoses of prosthetic endocarditis are compromised not only by local findings, but also by the inflammatory effects on the whole organism. Using a multicenter registry, Amat-Santos et al. demonstrated that the incidence of infective endocarditis 1 year after TAVI is 0.5%. The majority of affected patients from this registry were treated conservatively, and overall hospital mortality was approximately 47%, [14].

Overall, SAVR was associated with significant mortality in the patients included in our study, as expected. Even though none of the patients died on the operating table, i.e., the procedures were technically successful, the 30-day mortality was 45%. In 2013, Hein et al. published an almost identical 30-day mortality of 45.8% in patients who were converted from TAVI procedure to emergency cardiac surgery [15]. However, those authors studied only patients who required cardiac surgery immediately after TAVI; cardiac surgery at a later stage after TAVI was not considered in their publication. Furthermore, in contrast to our study, SAVR was not an explicit inclusion criterion in their registry analysis, and TAVI procedures with a primarily surgical approach were excluded from the outset.

Compared with data reported by Hein et al., our directly converted TAVI patients had a lower 30-day mortality of 33%. Two-stage procedures within 7 days of TAVI had a 30-day mortality of 67% in our collective, and we observed a 30-day mortality of 50% in subsequent valve surgeries after primary successful TAVI due to prosthetic endocarditis.

In our comparison of transfemoral and surgical TAVI procedures, we found that patients in whom the transfemoral approach was chosen had a better prognosis after SAVR. Indeed, only one patient with TF-TAVI access died within 30 days after SAVR. The remaining decedents had surgical access, i.e., two of them via the left ventricular apex and two via the ascending aorta. Extrapolating from these facts, our patients had an 80% 30-day survival after SAVR for subjects with primary TF-TAVI and of approximately 33% for those with primary surgical TAVI access. The lower survival of SAVR after surgical TAVI is certainly not due to the transapical or transaortic approach itself. Instead, it may be due to the fact that surgical TAVI patients have a different risk profile than those that are eligible for TF-TAVI [16]. In our collective, surgical TAVI patients had a higher EuroSCORE II, and findings requiring SAVR intraprocedurally or early in the course were more complex in such cases. Thus, only one patient required acute conversion after surgical TAVI because of a less complex prosthesis dislocation without concomitant cardiac problems. The failure of these interventions may be attributed to a wide range of factors.

According to our results, acute emergency surgery for primary unsuccessful TAVI is feasible with a reasonable risk, depending on the reason for conversion to SAVR. In cases of severe complications, such as circulatory collapse due physical occlusion of the left coronary main trunk that cannot be treated interventionally, the prognosis is very bad. In such cases, it may therefore be justified not to escalate treatment further. In contrast, in cases with only dislocated valve stent and existing circulatory stability, emergency SAVR may be carried out safely, because in this constellation, postoperative outcome seems to be acceptable, regardless of TAVI access. The 30-day mortality of these patients in our study was only 20%, and in individual cases, we observed survival times of 1 to almost 5 years.

In our experience, two-stage SAVR for early postoperative cardiac complications after primary successful TAVI is unpromising with regard to the mid-term prognosis.

In cases of late degenerative damage due to endocarditis of valve stents, we showed that SAVR is still associated with high risk. We also predict an acceptable chance for surgery on degenerated valvular stents, despite not having been assigned a patient with such characteristics for SAVR to date. Even if younger patients will more commonly undergo the TAVI procedure in the future, a significant number of such referrals is probably not to be expected, because, at most, mild and hemodynamically insignificant degeneration is observed on catheter valves 5 years after TAVI [16]. Moreover, subsequent problems not related to endocarditis, such as hemodynamically relevant degeneration or valvular and paravalvular insufficiencies, can, in principle, also be resolved by a repeat TAVI, [17].

## 5. Conclusions

Cases requiring emergency surgical intervention are most often those with improper TAV sizing and selection. Mostly, emergency cases may be attributed to valve dislocation either in the ascending aorta, aortic arch or LVOT. Such displacements either block the coronary ostia when moved in the direction of blood flow or lead to extensive aortic valve insufficiency when moved in the other direction. Those patients require an emergency thoracotomy and should be connected to a heart and lung machine. Also, TAV sizing mismatches play a significant role in cases requiring revision due to annulus rupture with or without VSD, sometimes even leading to the need for aortic dissection. Such cases must be also treated as life-threatening. In the study population, patients who presented in the late course with infective endocarditis also suffered from recurrent attacks of postoperative high-grade fever and shivering. Blood culture tests were mostly positive, and echocardiography proved the above diagnoses, and thus, the need for revision. The causes of SAVR after TAVI failure are multifactorial, including degenerative, physical or infectious factors. Acceptable mid-term prognoses were observed in patients with symptoms associated with the dislocation of the catheter prosthesis, for whom emergency conversions were required. Conversion due to infection, e.g., endocarditis, had the worst outcome. Prognoses after conversion due to degenerative causes are still lacking. This is due to a lack of enough autopsies and the recent history of prosthetic implantation. A proportion of affected patients can be saved by SAVR, both acute and subsequent. In cases of acute complications, rapid cardiac surgical intervention is required; in such instances, TAVI procedures must be performed at specialized centers with a broad cardiac surgical infrastructure. Cardiac surgery after TAVI should also be performed at TAVI centers with high levels of expertise, as these operations can be equally demanding.

### Limitations

Only patients who had undergone a TAVI procedure in our hospital and received a SAVR during the procedure or at a later stage were included. Since such operations are a rarity, very few patients could be studied. Therefore, the conclusions drawn here should be considered with caution.

## Figures and Tables

**Table 1:** Preoperative variables.

	Total (*n* = 11)	TF-TAVI (*n* = 5)	TA/TAO-TAVI (*n* = 6)
Male gender (*n*)	7 (64%)	2 (40%)	5 (83%)
Age (years)	79 (64–85)	78 (76–79)	83 (64–85)
EuroSCORE II	7.8 (1.8–35.3)	3.5 (1.8–7.8)	10.9 (7.2–35.3)
Previous cerebral insult (*n*)	2 (18%)	1 (20%)	1 (17%)
Coronary artery disease (*n*)	10 (91%)	5 (100%)	5 (83%)
Previous coronary stenting (*n*)	10 (91%)	5 (100%)	5 (83%)
Previous cardiac surgery (*n*)	0	0	0
Obstructive lung disease (*n*)	6 (55%)	1 (20%)	5 (83%)
Dialysis (*n*)	4 (36%)	1 (20%)	3 (50%)
Peripheral artery disease (*n*)	7 (64%)	2 (40%)	5 (83%)
Implant size (mm)	26 (23–34)	26 (23–34)	27.5 (23–34)

TF-TAVI: transfemoral transcatheter aortic valve implantation, TA/TAO-TAVI: transapical/transaortal transcatheter aortic valve implantation.

**Table 2:** TAVI-Metrics.

Patient	CAC-Score (Agatston Units)	Aortic Annulus (mm)	Sinotubular Junction (mm)	Distance between RCA Ostia to Annulus	Distance between LM Ostia to Annulus
Patient 1 (m, 80Y)	Mild	23 × 29 mm	n.m	20 mm	17 mm
Patient 2 (f, 78Y)	Severe	20 × 22 mm	n.m	14 mm	12 mm
Patient 3 (f, 76Y)	Extensive (2461)	20 × 25 mm	30 mm	20 mm	16 mm
Patient 4 (m, 83Y)	High	22 × 28 mm	29 mm	7 mm	16 mm
Patient 5 (m, 65Y)	High	23 × 34 mm	21 mm	13 mm	11 mm
Patient 6 (f, 83Y)	Mild	19 × 23 mm	22 mm	6 mm	13 mm
Patient 7 (m, 85Y)	Extensive (1955)	18 × 24 mm	29 mm	21 mm	16 mm
Patient 8 (m, 78Y)	Extensive (3028)	26 × 33 mm	29 mm	15 mm	8 mm
Patient 9 (f, 76Y)	Mild	17 × 24 mm	26 mm	15 mm	13 mm
Patient 10 (m, 83Y)	Extensive (2460)	25 × 29 mm	25 mm	17 mm	14 mm
Patient 11 (m, 79Y)	Extensive (1485)	22 × 28 mm	27 mm	16 mm	15 mm

CAC-Score: Coronary Artery Calcium score, n.m: no measurement, RCA: right coronary artery, LM: left main, f: Female, m: Male.

**Table 3:** TAVI prosthesis types and sizes.

	Total (*n* = 11)	TF-TAVI (*n* = 5)	TA/TAO-TAVI (*n* = 6)
Sapien XT^®^ (*n*)	2 (18%)	1 (20%)	1 (17%)
Sapien 3^®^ (*n*)	3 (27%)	1 (20%)	2 (33%)
CoreValve^®^ (*n*)	5 (45%)	2 (40%)	3 (50%)
Symetis^®^ (*n*)	1 (9%)	1 (20%)	0

**Table 4:** Core Data and Indications for Intervention.

Patient	TAVI	Problem	SAVR	30-Day Mortality
Patient 1 (m, 80Y)	TA-TAVI 26 mm Sapien XT	Dislocation into left ventricular outflow tract	Single-stage 27 mm Hancock II	no
Patient 2 (f, 78Y)	TF-TAVI 23 mm Sapien XT	Annulus perforation	≤7 days 21 mm PERIMOUNT	no
Patient 3 (f, 76Y)	TF-TAVI 26 mm CoreValve	Dislocation into ascending aorta	Single-stage 23 mm Hancock II circulatory arrest	no
Patient 4 (m, 83Y)	TAO-TAVI 29 mm CoreValve	Occlusion of the coronary left main trunk	Single-stage 21 mm Trifecta	yes
Patient 5 (m, 65Y)	TAO-TAVI 34 mm CoreValve	Paravalvular leakage	≤7 days 25 mm Hancock II ECLS before SAVR	yes
Patient 6 (f, 83Y)	TA-TAVI 26 mm Sapien 3	Annulus perforation with VSD	≤7 days 21 mm Trifecta closure of VSD	yes
Patient 7 (m, 85Y)	TAO-TAVI 29 mm CoreValve	Endocarditis	>3 months 25 mm Hancock II	no
Patient 8 (m, 78Y)	TF-TAVI 34 mm CoreValve	Dislocation into LVOT	Single-stage 29 mm Hancock II ECLS after SAVR	no
Patient 9 (f, 76Y)	TF-TAVI 25 mm Symetis	Dislocation into ascending aorta	Single-stage 25 mm Hancock II circulatory arrest	yes
Patient 10 (m, 83Y)	TA-TAVI 23 mm Sapien 3	Endocarditis	>3 months 21 mm Hancock II	yes
Patient 11 (m, 79Y)	TF-TAVI 26 mm Sapien 3	Dislocation into aortic arch	Single-stage 23 mm PERIMOUNT circulatory arrest	no

f: Female, m: Male., SAVR: surgical aortic valve replacement, ECLS: Extracorporeal Life Support System

**Table 5:** Timing of Surgery.

	Total (*n* = 11)	TF-TAVI (*n* = 5)	TA/TAO-TAVI (*n* = 6)
Single-stage operation (*n*)	6 (55%)	4 (80%)	2 (33%)
Two-stage ≤ 7 days (*n*)	3 (27%)	1 (20%)	2 (33%)
Two-stage > 3 months (*n*)	2 (18%)	0	2 (33%)
Catheter valve dislocation (*n*)	5 (45%)	4 (80%)	1 (17%)
Annulus perforation (*n*)	2 (18%)	1 (20%)	1 (17%)
Paravalvular leakage (*n*)	1 (9%)	0	1 (17%)
Ventricular septal defect (*n*)	1 (9%)	0	1 (17%)
Left main trunk occlusion (*n*)	1 (9%)	0	1 (17%)
Catheter valve endocarditis (*n*)	2 (18%)	0	2 (33%)

**Table 6:** Intraoperative Variables.

	Total (*n* = 11)	TF-TAVI (*n* = 5)	TA/TAO-TAVI (*n* = 6)
Bypass time (min)	122 (74–187)	122 (74–140)	127.5 (106–187)
Cross-clamp-time (min)	83 (49–143)	84 (49–102)	80 (72–143)
Circulatory arrest (*n*)	3 (27%)	2 (40%)	1 (17%)
Hancock II (*n*)	7 (64%)	3 (60%)	4 (67%)
PERIMOUNT^®^ (*n*)	2 (18%)	2 (40%)	0
Trifecta^®^ (*n*)	2 (18%)	0	2 (33%)
Implant size (mm)	23 (21–39)	23 (21–29)	23 (21–27)

**Table 7:** Postoperative Variables.

	Total (*n* = 11)	TF-TAVI (*n* = 5)	TA/TAO-TAVI (*n* = 6)
Stay on ICU (d)	5 (2–26)	3 (2–6)	9.5 (2–26)
Hospital length of stay (d)	15 (2–45)	15 (5–20)	17.5 (2–45)
Ventilation duration (h)	100 (15–283)	28 (15–125)	121 (48–283)
Tracheostomy (*n*)	2 (18%)	0	2 (33%)
Rethoracotomy (*n*)	2 (18%)	1 (20%)	1 (17%)
Delirium (*n*)	3 (27%)	0	3 (50%)
Cerebral Insult (*n*)	2 (18%)	1 (20%)	1 (17%)
Atrial fibrillation (*n*)	6 (55%)	3 (60%)	3 (50%)
Atrioventricular block (*n*)	4 (36%)	2 (40%)	2 (33%)
Pacemaker dependence (*n*)	1 (9%)	1 (20%)	0
Dialysis (*n*)	5 (45%)	1(20%)	4 (67%)
Wound infection (*n*)	1 (9%)	1 (20%)	0
30-day mortality (*n*)	5 (45%)	1 (20%)	4 (67%)
